# Motivations for (non)participation in population-based health studies among the elderly – comparison of participants and nonparticipants of a prospective study on influenza vaccination

**DOI:** 10.1186/s12874-017-0302-z

**Published:** 2017-02-02

**Authors:** Manas K. Akmatov, Leonhard Jentsch, Peggy Riese, Marcus May, Malik W. Ahmed, Damaris Werner, Anja Rösel, Jana Prokein, Inga Bernemann, Norman Klopp, Blair Prochnow, Thomas Illig, Christoph Schindler, Carlos A. Guzman, Frank Pessler

**Affiliations:** 1 TWINCORE, Centre for Experimental and Clinical Infection Research, Hannover, Germany; 2 Helmholtz Centre for Infection Research, Braunschweig, Germany; 3 Centre for Individualized Infection Medicine, Hannover, Germany; 4 MHH CRC Core Facility, Hannover Medical School, Hannover, Germany; 5 Hannover Unified Biobank, Hannover Medical School, Hannover, Germany; 6 Research Group “Biomarkers for Infectious Diseases”, TWINCORE, Centre for Experimental and Clinical Infection Research, Feodor-Lynen-Str. 7, Hannover, 30625 Germany

**Keywords:** Participation, Nonparticipants, Reasons, Motivations, Population-based study, Elderly, Germany

## Abstract

**Background:**

Participation in epidemiological studies has strongly declined in recent years. We examined the reasons for (non)participation in population-based health studies among participants and nonparticipants of a prospective study on influenza vaccination among the elderly.

**Methods:**

Males and females between 65 and 80 years of age (*N =* 5582) were randomly selected from the residents’ registration office in Hannover, Germany, and were invited to participate in a study featuring vaccination with a seasonal adjuvanted influenza vaccine (Fluad™, Novartis) including five follow-up visits (day 0, 1/3, 7, 21, 70 with respect to vaccination). A 24-item nonresponder questionnaire, including 10 items on reasons for participating in a hypothetical health study, was mailed to 1500 randomly selected nonparticipants. The same 10 items were included in the end-of-study questionnaire administered to the participants in the vaccination study (*n =* 200). Logistic regression analysis with backward elimination was used to identify the reasons most strongly associated with nonparticipation.

**Results:**

Five hundred thirty-one (35%) nonparticipants and 200 participants (100%) returned the respective questionnaires. Nonparticipation was associated with a lower interest in obtaining personal health information (OR = 3.32) and a preference for less invasive (OR = 3.01) and less time-demanding (OR = 2.19) studies. Responses to other items, e.g. regarding altruistic motives, monetary compensation, general interest of the study, or study approval through ethics committee and data security authority, did not differ between participants and nonparticipants.

**Conclusions:**

Participation rates in health studies among elderly individuals could potentially be improved by reducing interventions and time demand, for instance by implementing methods of self-sampling and remote data collection.

**Trial registration:**

No. 1100359 (ClinicalTrials.gov, date of registration: 09.02.2015).

**Electronic supplementary material:**

The online version of this article (doi:10.1186/s12874-017-0302-z) contains supplementary material, which is available to authorized users.

## Background

Participation in epidemiological studies has declined dramatically over the last few decades [1]. Any attempts to improve response rates and to reduce the resulting biases require an understanding of the reasons for (non)participation. A large body of research has examined various reasons for (non)participation in epidemiological studies [2, 3]. Most of the studies focused either on participants or nonparticipants, or compared the two groups only in terms of sociodemographic or health/disease differences such as sex, age, education level and presence of comorbidities [4, 5]. Apart from a comparison of sociodemographic factors between participants and nonparticipants, a direct comparison of motivations in these two groups leading to a decision to participate in a health-related study has not been made. In addition, very little is known about the reasons for (non)participation in health research among elderly individuals. Especially this population group possesses potentially important factors affecting willingness and/or ability to participate in health studies, such as overall increased morbidity with reduced physical mobility and time competition from other health-related or medical obligations. A few studies examined the reasons for (non)participation among elderly individuals in the context of specific topics. For example, Townsley et al. examined attitudes towards participation in clinical trials among 94 elderly cancer patients and found that recommendations from a cancer physician and personal benefit in form of better treatment were the most frequently reported reasons for participation [6]. Allsup and Gosney collected information on various reasons for nonparticipation in a randomized controlled trial on influenza vaccination among the 1173 elderly nonparticipants; the most frequently reported reasons were “unwillingness to participate in a research project” (53%), “concerns about side effects” (34%) and “do not require the vaccine” (32%) [7]. Costa et al. investigated motivations for participating in a trial on an avian influenza vaccine among 364 healthy adult participants [8]; the most important motivation for participating was an altruistic reason, where 43% reported “collaboration with science”. An altruistic reason for participating in a prospective study on Alzheimer’s disease was reported by half of the elderly study participants [9]. All of the above mentioned studies examined various reasons either among participants or among nonparticipants, but none of these studies compared, in parallel, the reasons stated by participants and nonparticipants of the same study.

While overall participation rates are dropping, epidemiological studies have been intensifying the depth of phenotyping, for instance by broadening the spectrum of medical examinations and by collecting biological samples such as saliva, blood, stool, or microbiological swabs for the establishment of biobanks for detailed molecular assessments [1]. Studies collecting less invasive biospecimens such as saliva may not influence the willingness to participate. However, less is known about studies that apply more invasive biospecimen collection associated with various risks of complications and more complex and intensified data collection protocols.

From September 2015 to May 2016, we conducted a population-based, prospective cohort study among individuals ≥65 years of age (*n =* 200), which featured vaccination with a seasonal influenza vaccine and collection of serial blood samples on days 0, 1/3, 7, 21 and 70. Applying a 10-item self-administered questionnaire on various reasons for participating in a hypothetical health study to both participants and nonparticipants of this study, we aimed to identify key factors leading to (non)participation of elderly individuals in health research.

## Methods

### Sampling and study design

Females and males between 65 and 80 years of age (*N =* 5582) were randomly selected by the residents’ registration office in Hannover, Germany. Invitations were sent by mail to participate in a prospective cohort study on influenza vaccination spanning the time period from September 2015 to May 2016. A travel reimbursement of 30 € was offered for participation. Individuals who agreed to participate were invited to the study center located in the Clinical Research Center (CRC) Hannover. The participants underwent medical examinations, including measurements of height, weight, and blood pressure, collection of a blood sample (75 ml) and subsequent influenza vaccination with an inactivated, trivalent, adjuvanted (MF59) vaccine (Fluad™, Novartis Vaccines and Diagnostics S.r.l., Rosia, Italy) licensed in Germany for use in individuals ≥65 years. Serial blood samples were collected on days 0, 1/3, 7, 21 and 70 after vaccination. A detailed description of the study protocol is provided elsewhere [10].

### Questionnaire on reasons for participating in health research

A 10-item self-administered paper-based questionnaire about reasons for participation in a hypothetical health study was administered to all participants in the vaccination study and randomly selected nonparticipants (see Additional file 1). All 10 items used the Likert scale with the five categories “very important”, “important”, “so-so”, “not important” and “not at all important”. In addition, there was one open-ended question. The questionnaire addressed the following reasons for (non)participation: altruistic motives [2 items], personal benefit (monetary and nonmonetary [2 items]), possible risks due to medical procedures such as blood samples [1 item], time demand [1 item], ethical and data security issues [2 items], participation in a study with an interesting topic [1 item], and seriousness of the study [1 item].

### Nonresponder survey

The nonparticipant group comprised individuals (1) who had notified us of their nonparticipation by returning a form included in the letter with the initial study invitation or (2) who did not respond to the initial study invitation. We conducted a nonresponder survey among 1500 (48% and 52% of male and females, respectively) randomly selected nonparticipants by sending them a 24-item questionnaire by mail, which included information on presence and frequency of upper and lower respiratory tract infections, gastrointestinal infections, presence of chronic diseases such as myocardial infarction, diabetes mellitus, cancer, stroke, history of influenza vaccinations, height and weight, self-perceived health status, education level, and the above-mentioned 10-item questionnaire on reasons for participating in health research.

### Statistical analyses

**Fig. 1:**
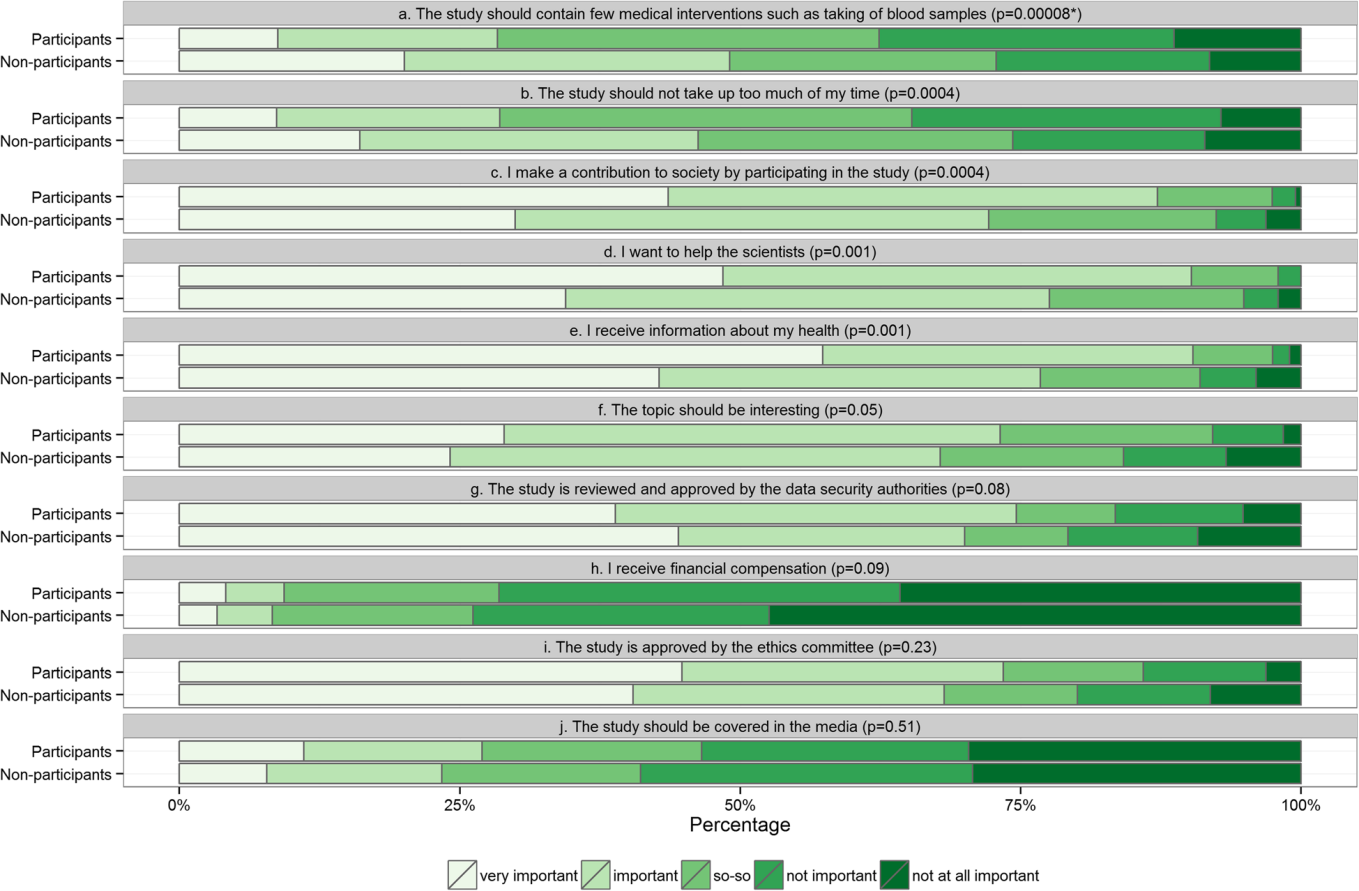
Reasons for participating in health research among participants and nonparticipants of the influenza vaccination study. * Chi-square test

**Fig. 2:**
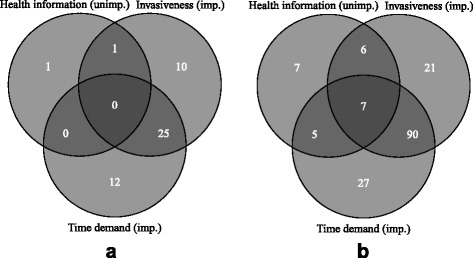
Venn diagrams of the relationships across the three reasons mostly associated with (non)participation in a hypothetical health study among participants (**a**) and nonparticipants (**b**) of the influenza vaccination study. The data are based on the number of individuals who responded with “very important” or “important” to the questions on time demand and medical interventions, and “not important” or “not at all important” to the question on receiving health information (see Fig. 1, Table 2). Abbreviations: imp. = important; unimp. = unimportant

The chi-square test was used to examine the significance of differences in the reasons for participating between participants and nonparticipants of the study. Furthermore, multivariable logistic regression analysis was used to identify the reasons significantly associated with nonparticipation. The outcome variable in a logistic regression model consisted of two categories: participants and nonparticipants of the influenza vaccination study. We modeled the probability of being a nonparticipant. Theoretically, the probability of being a participant can also be modeled, therefore, we used the term “(non)participation” that can be interpreted in both directions, i.e. reasons of participation or nonparticipation. All 10 items on reasons for participating and the variables sex, age (continuous variable), diabetes, cancer, myocardial infarction, self-perceived health status, and history of previous influenza vaccination were included in the model. The backward stepwise selection procedure based on the Wald test with a significance level of 0.1 for removal of the independent variables was applied [11]. We also performed a sensitivity analysis by excluding those nonparticipants whose main reason for not participating in the influenza vaccination study was that they had already received the vaccine for that season. Venn diagrams were used to address the question whether the three reasons most strongly associated with (non)participation were a characteristic common of specific subgroups of nonparticipants (and participants) or whether they were randomly distributed. Statistical analyses were carried out with IBM SPSS Statistics, version 20 (IBM Corporation, Armonk, NY, USA). Figures were made with the R Foundation for Statistical Computing (version 3.2.3); packages “ggplot2” and “VennDiagram” were used for Fig. 1 and Fig. 2, respectively.

## Results

Of the 5582 invited individuals, 223 agreed to participate in the influenza vaccination study, resulting in an initial response rate of 4.0%. After screening for the exclusion criteria, 181 individuals were eligible to participate, corresponding to a final participation rate of 3.2% (181/5582). In addition, we included 19 individuals from a convenience sample (mostly spouses of study participants). Thus, the final study population consisted of 200 individuals, all of whom completed the end-of-study questionnaire. Of the 1500 nonparticipants, 531 (35%) returned the nonresponder questionnaire.

### Differences between participants and nonparticipants

There were significant differences between the participants and nonparticipants of the study in terms of diabetes mellitus and self-perceived health status (Table 1); participants differed from nonparticipants by less self-reported diabetes mellitus (*X* ^*2*^ = 7.10, df = 1, *n =* 716, *p =* 0.008) and a better self-perceived health status (*X* ^*2*^ = 24.64, df = 4, *n =* 680, *p <* 0.0001). There were no significant differences in terms of sex, age, self-reported myocardial infarction, cancer, and history of previous influenza vaccination (Table 1).

**Table 1:** Comparison of the participants and nonparticipants of the influenza vaccination study in terms of demographic and health-related variables, %

	Participants of the study (*n =* 200)	Nonparticipants of the study (*n =* 531)	*p* value*
Sex			0.08
Female	43	50	
Male	57	50	
Median age in years (interquartile range)	72 (68–76)	73 (69–76)	0.11^a^
Body Mass Index^b^			0.37
Underweight (≤18.49 kg/m^2^)	1.0	1.6	
Normal weight (18.50-24.99 kg/m^2^)	36	42	
Overweight (25.00-29.99 kg/m^2^)	45	38	
Obesity (≥30.00 kg/m^2^)	18	18	
Myocardial infarction			0.68
Yes	6.0	6.8	
No	93	91	
Don’t know	0.50	0.38	
Missing values	0.50	1.7	
Cancer			0.54
Yes	20	22	
No	80	76	
Don’t know	0	1.1	
Missing values	0	1.7	
Diabetes mellitus			0.008
Yes	7.5	15	
No	92	83	
Don’t know	0.50	0.57	
Missing values	0	1.7	
Self-perceived health status			<0.0001
Poor	0	0.19	
Fair	9.0	15	
Good	64	62	
Very good	21	14	
Excellent	4.0	0.19	
Missing values	2.0	8.0	
Ever vaccinated against influenza			0.93
Yes	78	78	
No	21	21	
Don’t know	1.0	0.19	
Missing values	1.5	1.1	

*Chi-square test, the category “don’t know” and missing values were not considered in this test

^a^Mann–Whitney test

^b^Body Mass Index (BMI) was calculated using the formula “weight/height^2^” (kg/m^2^). Weight and height were objectively measured at the study center in the influenza vaccination study and self-reported by the nonparticipants

### Reasons for nonparticipation in the influenza vaccination study

The reported reasons for not participating in the study were “I have already been vaccinated” (55%), “I was not convinced of aim and purpose of the study” (9.7%), “no specific reason” (8.8%), “due to lack of time” (8.0%), “for health reasons” (7.4%), “no interest” (6.3%), “too many blood draws” (3.2%), “for employment-related reasons” (1.1%), and “language problems” (0.84%).

### Reasons for (non)participation in health research

In a univariable analysis we found six reasons significantly different between the participants and nonparticipants (Fig. 1). For example, around 20% of the nonparticipants reported that “The study should contain few medical interventions such as taking blood sample” compared to only 8.8% of the participants. Around 16% of the nonparticipants stated that “The study should not take up too much of my time” compared to only 8.7% of the participants. In a multivariable analysis five reasons remained in the model after backward elimination (Table 2). The strongest association was observed for the item “I receive information about my health”; individuals, who reported “not important” on this item, were more likely not to participate. In addition, individuals who reported importance with “Few medical interventions such as blood collection” and “The study should not take up too much of my time” were more likely to be nonparticipants. Also, females, individuals with self-reported diabetes and with poor/fair self-perceived health status were more likely not to participate (Table 2). The final model with the remaining items explained 24% of the variance (Nagelkerke’s pseudo R^2^: 0.24). In the sensitivity analysis (in which individuals were excluded who had stated prior vaccination as the reason for not participating) the effects of the three items mentioned above increased (Table 2, sixth column). In addition, two items “Review and approval by the data security authorities” and “A contribution to society” were significantly associated with being a nonparticipant (Table 2, sixth column).

**Table 2:** Effect of motivations to participate in health research on nonparticipation in the influenza vaccination study among elderly individuals (results of multivariable logistic regression analyses)

	Total sample^a^			Subsample^a b^		
Items	*n*	AOR (95% CI)^c^	*p* value	*n*	AOR (95% CI)^c^	*p* value
The study should contain few medical interventions such as taking of blood samples.						
important	183	3.01 (1.71–5.31)	<0.0001	108	3.53 (1.71–7.26)	0.001
so-so	127	0.99 (0.57–1.73)	0.98	93	1.29 (0.62–2.70)	0.50
not important	141	Ref.		99	Ref.	
The study should not take up too much of my time.						
important	175	2.19 (1.27–3.78)	0.005	103	2.77 (1.34–5.70)	0.006
so-so	143	1.27 (0.74–2.18)	0.39	105	1.62 (0.78–3.37)	0.20
not important	133	Ref.		92	Ref.	
I receive information about my health.						
important	368	Ref.		241	Ref.	
so-so	56	2.50 (1.20–5.20)	0.01	39	2.58 (1.08–6.15)	0.03
not important	27	3.32 (1.12–9.81)	0.03	20	3.44 (1.01–11.77)	0.049
The study is reviewed and approved by the data security authorities.						
important	324	Ref.		210	Ref.	
so-so	42	2.45 (1.08–5.55)	0.03	29	3.08 (1.18–8.05)	0.02
not important	85	1.54 (0.84–2.82)	0.10	61	2.14 (1.02–4.52)	0.045
I make a contribution to society by participating in the study.						
important	347	Ref.		223	Ref.	
so-so	79	2.43 (1.29–4.59)	0.006	57	3.90 (1.89–8.03)	<0.0001
not important	25	2.54 (0.86–7.49)	0.09	20	4.95 (1.55–15.77)	0.007
Sex						
Female	205	1.87 (1.20–2.91)	0.006	122	1.82 (1.02–3.22)	0.04
Male	246	Ref.		178	Ref.	
Diabetes mellitus						
Yes	59	1.82 (0.90–3.66)	0.10	42	2.27 (1.03–4.99)	0.04
No	392	Ref.		258	Ref.	
Self-perceived health status						
Poor/fair	83	3.53 (1.54–8.09)	0.003	62	4.00 (1.42–11.30)	0.009
Good	310	2.07 (1.19–3.59)	0.01	202	2.30 (1.12–4.73)	0.02
Very good/excellent	58	Ref.		36	Ref.	

*AOR* adjusted odds ratio, *CI* confidence intervals

^a^Backward elimination procedure based on the Wald test was applied to create the final model

^b^The nonparticipants whose main reason for nonparticipating in the influenza vaccination study was having been already vaccinated against influenza were excluded (see sensitivity analysis in the Methods section)

^c^Adjusted for all other variables in the table

The Venn diagrams in Fig. 2 show the numbers of participants and nonparticipants who answered “very important” or “important” to the questions relating to time demand and medical interventions, and “not important” and “not at all important” to the question relating to health information. In both groups there was an association between the questions on time demand and medical interventions, in that 58% of all participants and 52% of all nonparticipants who gave importance to one also did so to the other. On the other hand, there were no participants and only 4% nonparticipants who, in addition, stated that information on personal health would not be an important reason for participation.

## Discussion

To the best of our knowledge, this is the first study which, by applying a questionnaire in parallel to both participants and nonparticipants, aimed to identify reasons for (non)participation in health research among participants and nonparticipants of a population-based health-related study among the elderly. Previous research examined the reasons either in qualitative studies with small groups of individuals [12–14] or were of a descriptive nature by recording the reasons either only among participants [8] or nonparticipants [7, 15]. In contrast to those studies, we used an analytical approach by applying multivariate techniques with automatic selection to identify the most important reasons for (non)participation. In contrast to other studies, the participants and nonparticipants of our influenza vaccination study did not differ much in terms of basic sociodemographic and health-related characteristics such as age, Body Mass Index, self-reported myocardial infarction and cancer. However, multivariable analyses showed that the risk of nonparticipation increased significantly among females, those with diabetes, and a poorer self-perceived health status, indicating a possible selection bias.

The identified reasons for (non)participation in health research were the combination of various reasons and can be summarized into three groups: a) reasons associated with personal benefit (e.g. receiving information about personal health status), b) reasons associated with invasiveness of the study instruments (e.g. blood draws), and c) reasons associated with time demand by the study. In addition, altruistic motives and data security issues were significantly associated with (non)participation, but only in the sensitivity analysis. The latter analysis excluded the nonparticipants whose main reason for not participating in the influenza vaccination study was that they had already received the vaccine for that season. The remaining nonparticipants may be considered “true” nonparticipants and the two items identified in the sensitivity analysis (altruistic motives and data security issues) should therefore also be taken into account.

Personal benefit in form of monetary and nonmonetary advantage has been observed in many studies and is used as an incentive to increase response rates [16]. Receiving information on personal health was found to be a driving reason for participation in genomic studies among elderly individuals in Switzerland [17]. Benefits from participation in a health study should be clearly communicated to potential study participants to increase the response rates, e.g. through addressing them adequately in the consent forms. In line with our finding on time demand by the study, Gaertner et al. observed that “no time” was among the top five reasons for nonparticipation reported in a health survey among elderly nonparticipants in Berlin, Germany [15]. Alternative methods of data collection that reduce the time demands by a study should be considered, such as home visits, self-collection of biosamples, or web-based data collection. However, web-based data collection might not be feasible in the current elderly population. This might change considerably in the future, as a result of aging of computer/internet users and increasing acceptance of electronic media among senior citizens.

There was considerable overlap between the subgroups who considered low time demand and low number of medical interventions important (see Fig. 2). This observation makes sense from the point of view of human behavior, as “impatient” personality traits or perceived or real high demands on an individual’s time may likely affect the responses to these two questions similarly. On the other hand, there was only a very small percentage of nonparticipants who, in addition, did not find receiving health information a motivating factor. Thus, we did not identify a specific subgroup of individuals with particularly adverse attitudes toward participating in health research.

A monetary incentive turned out to not be a determinant of (non)participation. This is in line with results from our other population-based studies in urban Northern Germany, i.e. a resource-rich setting. In a pilot study in Pretest 2 of the German National Cohort, a monetary incentive was not associated with participants’ willingness to participate in future studies [18]. Likewise, in a study on serial home-collection of anterior nasal swabs for *S. aureus* surveillance, a monetary incentive did not increase participants’ compliance with the study protocol [19]. These findings are important for cost-efficient planning of future studies in populations such as ours, but may not be transferable to research settings in resource-poor countries.

The response rate to the influenza vaccination study was very low. Therefore, new strategies are required to recruit adequate numbers of study participants for studies where representativeness of the general population is important. Sampling based on the residents’ registry alone turned out to be an inefficient way to recruit a study population representative of the general aging population. Although we oversampled older age groups to achieve representativeness in terms of age, it is difficult to control for other parameters. For example, we missed frail individuals living in nursing homes. Thus, one needs to consider other sampling approaches in addition to the traditional register-based sampling, such as recruitment in nursing or residential care homes for elderly individuals, family physicians’ offices, or other places with a high accumulation of senior citizens, such as senior citizens’ associations or community support structures for the elderly. A recent systematic review showed that response rates were higher among population-based studies which apply face-to-face interviews, provide home visits for examinations, or had a less intensive study protocol [20]. We did not address the first two aspects in our present study, but it appears plausible that, in addition to offering a stream-lined study protocol with few interventions, providing home visits could further increase participation rates in health-related studies among elderly individuals.

### Limitations of the study

This study has two potential limitations. First, only one third of the nonparticipants returned the nonresponder questionnaire. This subsample may be biased in terms of selected sociodemographic characteristics and may thus not be representative of the general population. For instance, we failed to obtain information about nonparticipants who could not complete and/or mail the questionnaire due to frailty or dementia. Second, the 10-item questionnaire was administered to the participants of the vaccination study at the last follow-up visit. Experiences made while participating in the study may have affected their attitudes toward health research and participation in health-related studies.

## Conclusions

We identified a low interest in obtaining personal health information and a preference for less intrusive and time demanding studies as major determinants of nonparticipation in health-related studies among the elderly. These findings may aid in adapting study protocols to improve participation rates for future population-based health studies, particularly among the elderly.

## Additional file

Additional file 1: Questionnaire on reasons to participate in health research. (PDF 125 kb)

## Abbreviations

AOR: Adjusted odds ratio
BMI: Body mass index
CI: Confidence interval
CRC: Clinical research centre
imp: Important
unimp: Unimportant
