# Supplementary material for: Motivations for (non)participation in population-based health studies among the elderly – comparison of participants and nonparticipants of a prospective study on influenza vaccination
Source: BMC Med Res Methodol. 2017 Feb 2;17:18. doi: 10.1186/s12874-017-0302-z (PMC5288977; doi:10.1186/s12874-017-0302-z)
Supplement: Additional file 1: — Questionnaire on reasons to participate in health research. (PDF 125 kb) [file 12874_2017_302_MOESM1_ESM.pdf]

Questionnaire on reasons to participate in health research

If you think of participating in a health study, which reasons are important for your decision to participate?

Wenn Sie sich überlegen, an einer Studie zur Gesundheit teilzunehmen, wie wichtig sind die folgenden Gründe für Ihre Entscheidung?

English	German	very important	important	so-so	not important	not at all important
I receive financial compensation.	Ich erhalte eine finanzielle Aufwandsentschädigung.	☐	☐	☐	☐	☐
The topic should be interesting.	Es sollte ein interessantes Thema sein.	☐	☐	☐	☐	☐
The study should be covered in the media.	Von der Studie wird in den Medien berichtet.	☐	☐	☐	☐	☐
I want to help the scientists.	Ich will den Wissenschaftlern helfen.	☐	☐	☐	☐	☐
I receive information about my health.	Ich erhalte Informationen zu meiner Gesundheit.	☐	☐	☐	☐	☐
The study is approved by the ethics committee.	Die Studie wird von der zuständigen Ethikkommission genehmigt.	☐	☐	☐	☐	☐
The study is reviewed and approved by the data security authorities.	Die Studie wird von dem zuständigen Datenschützer überprüft und genehmigt.	☐	☐	☐	☐	☐
The study should contain few medical interventions such as taking of blood samples.	Die Studie soll wenig medizinische Eingriffe wie z.B. Blutentnahmen haben.	☐	☐	☐	☐	☐
The study should not take up much of my time.	Die Studie nimmt nicht viel Zeit in Anspruch.	☐	☐	☐	☐	☐
I make a contribution to society by participating in the study.	Mit meiner Studienteilnahme leiste ich einen Beitrag für die Gesellschaft.	☐	☐	☐	☐	☐
		**sehr wichtig**	**eher wichtig**	**teil-teils**	**eher nicht wichtig**	**gar nicht wichtig**
